# Cancer of the prostate presenting with diffuse osteolytic metastatic bone lesions: a case report

**DOI:** 10.1186/1752-1947-6-425

**Published:** 2012-12-28

**Authors:** Innocent Lule Segamwenge, Nuru Kaddu Mgori, Safia AbdallahYussuf, Celia Nantume Mukulu, Philip Nakangombe, Paul Kioko Ngalyuka, Fred Kidaaga

**Affiliations:** 1 Department of Internal Medicine, Intermediate Hospital Oshakati, Private Bag 5501, Oshakati, Namibia; 2 Department of Radiology, Intermediate Hospital Oshakati, Oshakati, Namibia; 3 Department of Urology, Intermediate Hospital Oshakati, Oshakati, Namibia; 4 Namibian Institute of Pathology, Khomas, Namibia

**Keywords:** Bone, Metastases, Osteolytic, Prostate cancer

## Abstract

**Introduction:**

Prostate cancer is the second most common cancer in men and the fifth most common cancer worldwide. In the USA it is more common in African-American men than in Caucasian men. Prostate cancer frequently metastasizes to bone and the lesions appear osteoblastic on radiographs. Presentation with diffuse osteolytic bone lesions is rare. We describe an unusual presentation of metastatic prostate cancer with diffuse osteolytic bone lesions.

**Case presentation:**

A 65-year-old Namibian man presented with anemia, thrombocytopenia and worsening back pains. In addition he had complaints of effort intolerance, palpitations, dysuria and mild symptoms of bladder outlet obstruction. On examination he was found to be anemic, had a swollen tender right shoulder joint and spine tenderness to percussion. On digital rectal examination he had asymmetrical enlargement of the prostate which felt nodular and hard with diffuse firmness in some parts. His prostate-specific antigen was greater than 100ng/mL and he had diffuse osteolytic lesions involving the right humerus, and all vertebral, femur and pelvic bones. His screen for multiple myeloma was negative and the prostate biopsy confirmed prostate cancer.

**Conclusion:**

Prostate cancer rarely presents with diffuse osteolytic bone lesions and should be considered in the differential diagnosis when evaluating male patients with osteolytic bone lesions.

## Introduction

Prostate cancer is the second most frequently diagnosed cancer of men after lung cancer and the fifth most common cancer worldwide [1]. It constitutes 10.6% of cancers of men in sub-Saharan Africa [2]. In the USA the tumor appears to be more common in African-American men than in Caucasian men [3].

Prostate cancer frequently metastasizes to bone but metastases can also be found in other body organs and tissues [4]. They contribute significantly to the morbidity associated with advanced disease [5]. The bone metastases of prostate cancer are usually radiologically osteoblastic. Presentation with diffuse osteolytic bone metastases is rare and only a few case reports exist in the medical literature with this type of metastatic lesion [6,7]. We present an unusual presentation of prostate cancer with osteolytic bone metastases.

## Case presentation

A 65-year-old Namibian man was referred to our hospital by a district hospital for evaluation of anemia, thrombocytopenia and back pain. The patient had been in his usual state of health until seven months prior to admission when he started experiencing low back pain. The pain was initially mild but gradually increased in intensity to severe pain by the time of admission. The pain was radiating to the patient’s lower limbs; it worsened with routine activities like walking, and was relieved by rest. There was no associated trauma to the back. In addition, the patient complained of exertional dyspnea, fatigue and palpitations four months prior to admission.

A review of his other systems revealed complaints of weight loss, fevers and general malaise. He also complained of straining while passing urine and frequency of micturition and dysuria. He had neither symptoms of cough, difficulty in breathing nor symptoms related to the gastrointestinal tract. His past medical and surgical history were unremarkable. The patient did not consume alcohol or smoke cigarettes.

**Figure 1:**
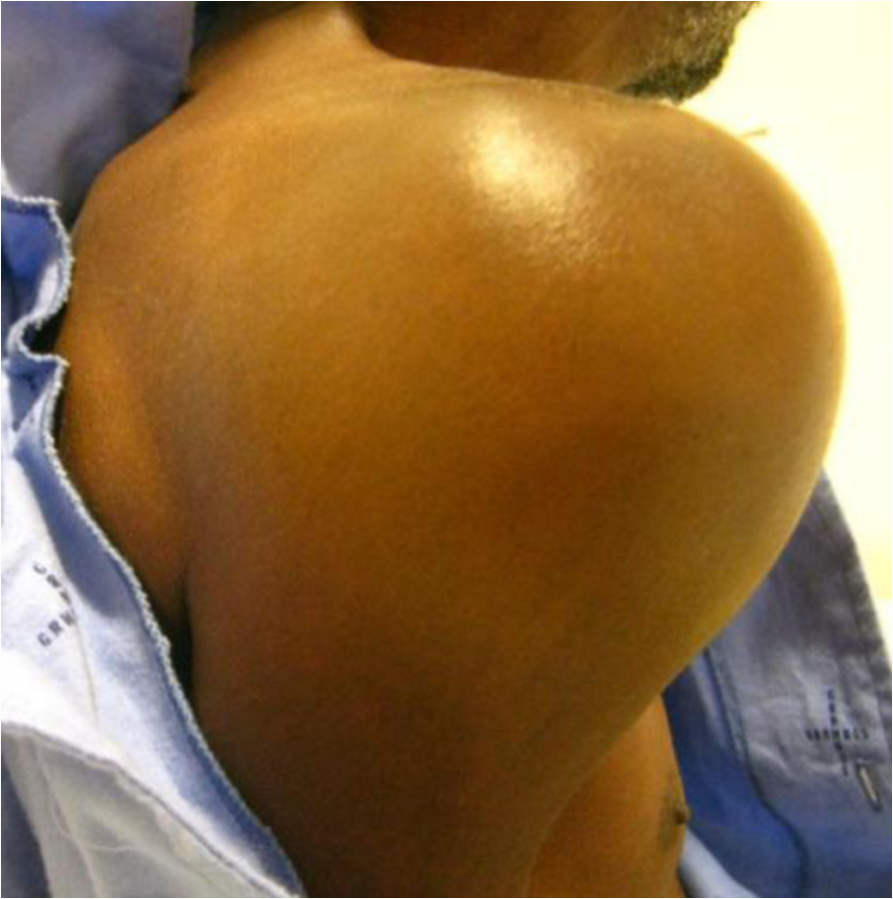
Swollen right shoulder joint.

On examination the patient had moderate pallor and mild wasting. The right shoulder joint was swollen and tender with limited range of movement (Figure 1). He had tenderness to percussion over the thoracic and lumbar spines with no swelling or deformity noted. On digital rectal examination he had asymmetrical enlargement of the prostate which felt nodular and hard with diffuse firmness in some parts. Other examination findings were normal.

The patient had a normocytic normochromic anemia of 5.4g/dL and thrombocytopenia of 42,000/μL. The absolute reticulocyte count was 101,500 cells/μL and a reticulocyte index of 1.1. The peripheral blood smear showed anisocytosis, polychromasia, nucleated red cells, no rouleaux formation and the platelet morphology was normal. Other blood tests showed hypercalcemia of 2.9mmol/L, raised alkaline phosphatase (ALP) of 509IU/L and lactate dehydrogenase (LDH) of 547IU/L. The renal function tests and liver function tests were normal. The serum protein electrophoresis showed no M-protein band and the urine was negative for Bence Jones proteins.

The urine analysis showed mild proteinuria of 30mg/dL with microscopic hematuria and the urine culture grew *Escherichia coli* sensitive to cefuroxime. The prostate-specific antigen (PSA) was greater than 100ng/mL.

**Figure 2:**
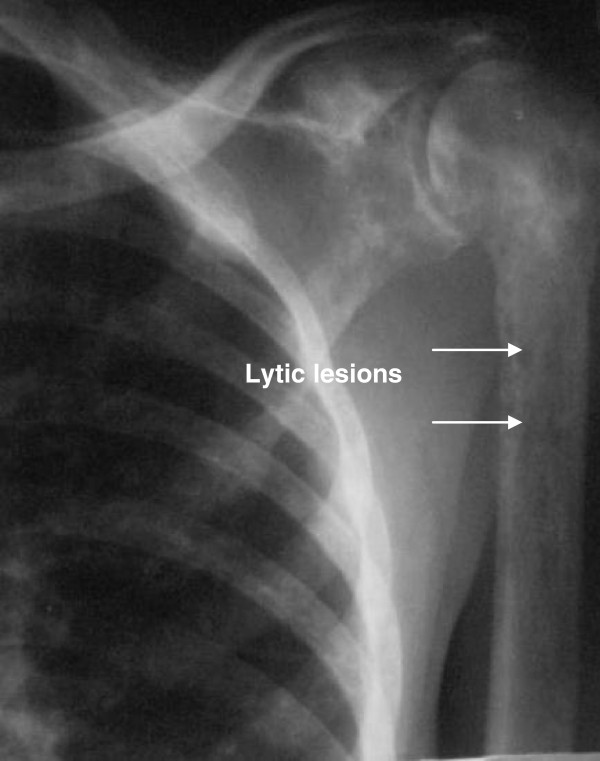
Lytic lesions (arrows) involving the humerus.

**Figure 3:**
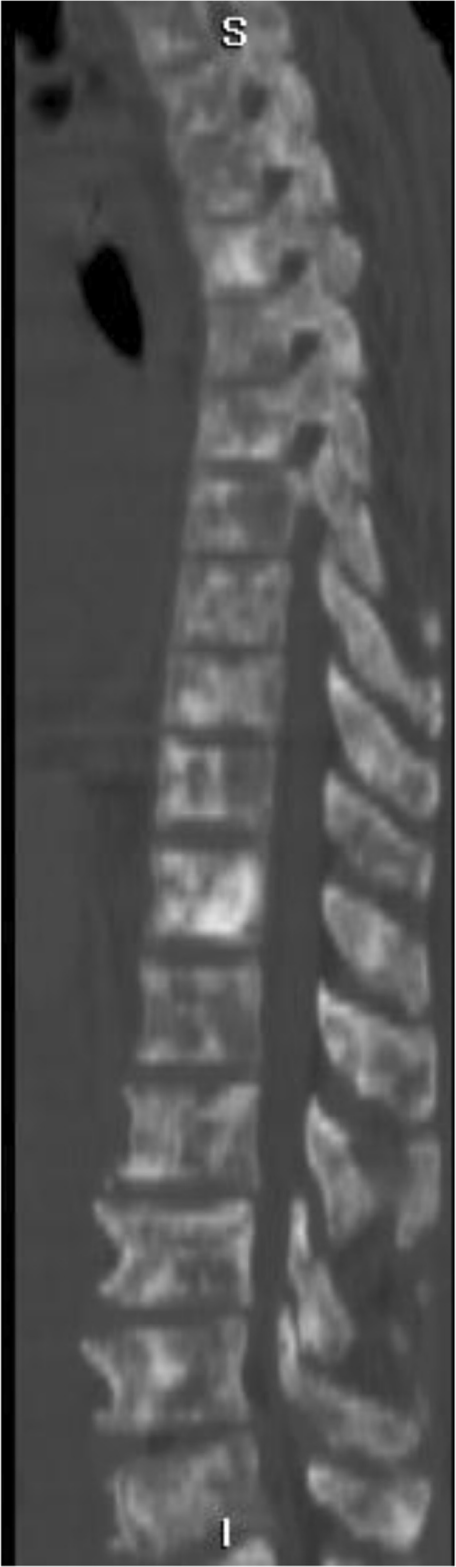
Computed tomography scan showing lytic lesions involving the entire spine.

**Figure 4:**
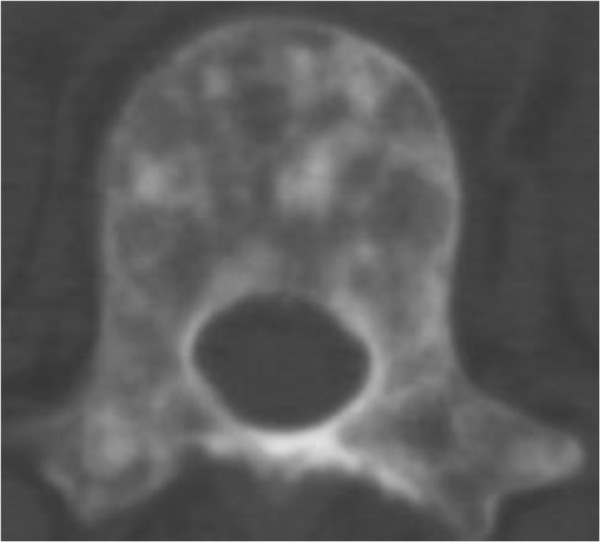
Thoracic vertebrae with lytic lesions.

**Figure 5:**
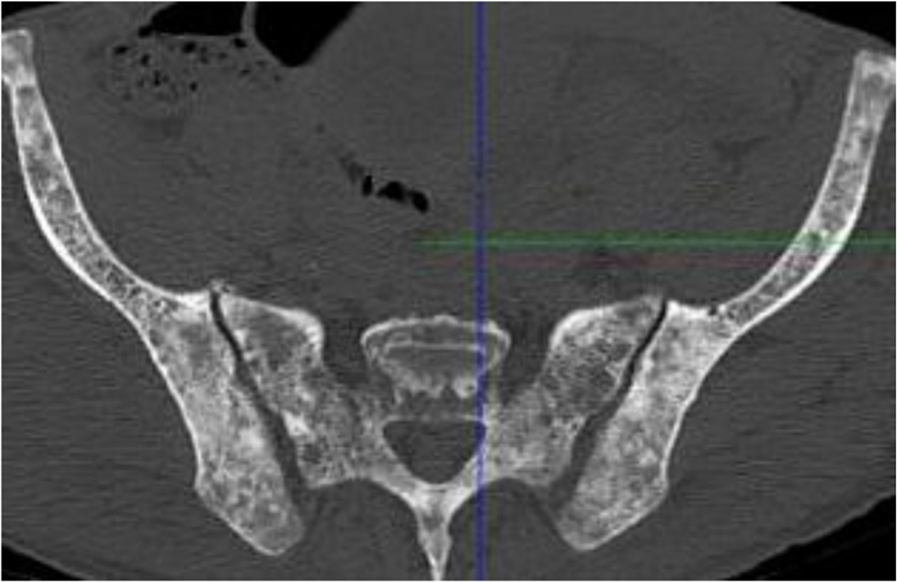
Sacral and pelvic bones with lytic lesions.

**Figure 6:**
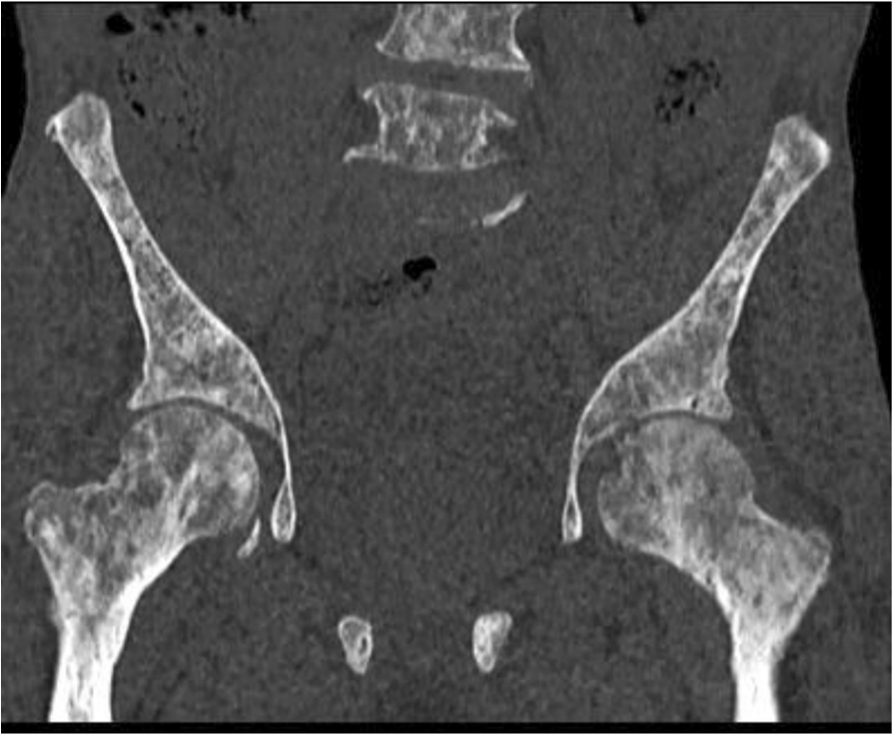
Computed tomography scan showing lytic lesions involving the femur and pelvic bones.

X-rays of the patient’s right shoulder joint showed osteolytic bone lesions on his humerus (Figure 2). Similar lesions were seen on computed tomography scans of his lumbar spine, femur and pelvic bones (Figures 3, 4, 5 and 6). The lateral skull X-ray was normal.

A biopsy of the prostate was done and the subsequent histology confirmed infiltrating adenocarcinoma of the prostate with a Gleason score of 9.

A bone marrow trephine showed a diffuse infiltrate of metastatic adenocarcinoma which stained positive to PSA immunohistostain confirming the prostatic origin of the metastases.

The patient received a blood transfusion and his hemoglobin level was raised to 10g/dL. The urinary tract infection was treated with a seven-day course of cefuroxime. He was given adequate pain relief with 100mg of oral tramadol per eight hours. The patient declined to have bilateral orchidectomy the only form of androgen deprivation therapy available in the Namibian free health scheme. He continues to receive palliative care.

## Discussion

Prostate cancer frequently metastasizes to bone with up to 90% of patients with advanced disease having bone involvement [8,9]. Bone metastases contribute significantly to the morbidity associated with advanced cancer of the prostate such as bone pain, immobility, pathological fractures, hypercalcemia, hematological disorders and spinal cord compression [10]. It has been shown that prostate cancer metastasizes to the spine before involvement of other organs [8]. Our patient had bone metastases as the initial presentation of disease with severe bone pains, hypercalcemia and hematological complications of anemia and thrombocytopenia.

The bone metastases of prostate cancer have a characteristic osteoblastic appearance on radiographs. Metastatic presentation with osteolytic lesions is rare with only a few case reports in the scientific literature describing this presentation [6,7]. This type of presentation may lead to delayed diagnosis because patients will be investigated for other causes of osteolytic bone lesions especially multiple myeloma. Our patient was 65 years of age, had bone pains, anemia and thrombocytopenia which could all have supported a diagnosis of multiple myeloma. In addition, the patient did not present with significant symptoms of bladder outlet obstruction which may otherwise have led us to suspect the prostate as the primary site. When evaluated for multiple myeloma, he had no lytic lesions in the skull bones or mandible and serum electrophoresis was negative for M-protein and there were no Bence Jones proteins in the urine. A bone marrow biopsy excluded the possibility of a non-secretory form of multiple myeloma. The presence of metastatic prostate cancer in the lesions was confirmed by the positive staining with PSA immunohistostain.

Our patient had a PSA level greater than 100ng/mL and an elevated ALP level of 509IU/L. PSA levels greater than 20ng/mL and ALP levels greater than 90IU/L have been found to be predictors for the presence of bone metastases among patients with prostate cancer [11]. The PSA is thought to play a role in the genesis of osteoblastic bone lesions by promoting the proliferation of osteoblasts and apoptosis of osteoclasts. With such an elevated PSA level our patient would have been expected to have predominantly osteoblastic lesions.

The pathogenesis of bone lesions in prostate cancer is not well understood. Prostate cancer cells promote both osteolytic and osteoblastic activity through production of factors that have direct or indirect osteogenic properties [12]. Factors such as bone morphogenetic proteins, endothelin-1, PSA and parathyroid hormone-related protein promote osteoblastic activity. The receptor activator of nuclear factor kappa-B ligand (RANKL) and its receptor (RANK) signaling promote osteoclastic activity while osteoprotegerin (OPG) protects the skeleton from excessive bone resorption by binding to RANKL and preventing it from binding to its receptor, RANK [13]. Prostate cancer cells express OPG and RANKL [14]. The RANKL to OPG ratio determines bone mass with a decrease in OPG resulting in excessive bone resorption. It is possible that in some patients with predominantly osteolytic bone lesions the RANKL to OPG balance is altered and hence the appearance of osteolytic bone lesions as was seen in our patient.

The treatment modalities of advanced prostate cancer are all palliative. Our patient received a blood transfusion and adequate pain relief medications. Androgen deprivation therapy is the standard of care for palliative treatment of prostate cancer [15]. It comprises surgical castration or suppression of luteinizing hormone-releasing hormone (LHRH) production. Pharmacological castration with diethylstilbestrol or LHRH agonists achieves testosterone levels similar to surgical castration; however, these agents are expensive and not readily available to most patients in low resource settings. Surgical castration may sometimes not be psychologically acceptable as was the case in our patient.

## Conclusion

Prostate cancer rarely presents with osteolytic bone lesions and should be considered in the differential diagnosis when evaluating male patients with osteolytic bone lesions.

## Consent

Written informed consent was obtained from the patient for publication of this case report and accompanying images. A copy of the written consent is available for review by the Editor-in-Chief of this journal.

## Abbreviations

ALP: Alkaline phosphatase; LHRH: Luteinizing hormone-releasing hormone; OPG: Osteoprotegerin; PSA: Prostate-specific antigen; RANK: Receptor activator of nuclear factor kappa-B; RANKL: Receptor activator of nuclear factor kappa-B ligand.

## Competing interests

The authors declare that they have no competing interest.

## Authors’ contributions

ILS conceived the idea and wrote the manuscript. NKM, ILS, SY, CM, PN and PKN investigated and treated the patient. FK confirmed the presence of metastatic prostate cancer in bone lesions. The final manuscript was read and approved by all authors.
